# A dataset of dual calcium and voltage optical mapping in healthy and hypertrophied murine hearts

**DOI:** 10.1038/s41597-021-01085-5

**Published:** 2021-12-16

**Authors:** Shicheng He, Kun Kou, Christopher O’Shea, Tangting Chen, Razik Mu-u-min, Ruirui Dong, Huiying Ren, Xiaolin Zhou, Zhongcai Fan, Xiaoqiu Tan, Davor Pavlovic, Xianhong Ou, Ming Lei

**Affiliations:** 1 Key Laboratory of Medical Electrophysiology of Ministry of Education and Medical Electrophysiological Key Laboratory of Sichuan Province, Institute of Cardiovascular Research, Southwest Medical University, Luzhou, China; 2 Department of Cardiovascular Medicine, The Affiliated Hospital of Southwest Medical University, Luzhou, China; 3 Institute of Cardiovascular Sciences, University of Birmingham, Birmingham, United Kingdom; 4 Department of Pharmacology, University of Oxford, Oxford, United Kingdom

**Keywords:** Imaging and sensing, Cardiac hypertrophy

## Abstract

Pathological hypertrophy underlies sudden cardiac death due to its high incidence of occurrence of ventricular arrhythmias. The alteration of transmural electrophysiological properties in hypertrophic cardiac murine tissue has never been explored previously. In this dataset, we have for the first time conducted high-throughput simultaneous optical imaging of transmembrane potential and calcium transients (CaT) throughout the entire hypertrophic murine hearts at high temporal and spatial resolution. Using ElectroMap, we have conducted multiple parameters analysis including action potential duration/calcium transient duration, conduction velocity, alternans and diastolic interval. Voltage-calcium latency was measured as time difference between action potential and CaT peak. The dataset therefore provides the first high spatial resolution transmural electrophysiological profiling of the murine heart, allowing interrogation of mechanisms driving ventricular arrhythmias associated with pathological hypertrophy. The dataset allows for further reuse and detailed analyses of geometrical, topological and functional analyses and reconstruction of 2-dimensional and 3-dimentional models.

## Abstract (summary)

**Table:** 

Measurement(s)	transmembrane potential • calcium transient
Technology Type(s)	high-throughput simultaneous optical imaging
Sample Characteristic - Organism	Mus musculus

Machine-accessible metadata file describing the reported data: 10.6084/m9.figshare.16910542

## Background & Summary

Sudden cardiac death (SCD) due to ventricular arrhythmias causes 3.7 million deaths worldwide every year^1^. SCD is highly associated with pathological hypertrophy though it also occurs in a broad spectrum of other cardiac conditions. Electrophysiological studies over the past few decades in animal models were largely based on the isolated single cell preparations. Nevertheless, these studies enabled us to gain a better understanding of how pathological cardiac remodelling may predispose to ventricular arrhythmias that may lead to SCD. However, even in healthy heart, a remarkable regional and transmural differences in electrophysiological properties and expression of ion channels exists across the myocardium. Therefore, it is necessary to develop a robust technique for studying transmural electrophysiological properties in living cardiac tissue in order to better understand drivers of cardiac arrhythmias.

Recently we developed a high-throughput transverse slice optical imaging technique as a new approach for studying cellular electrophysiology of murine heart in intact sliced ventricular tissue. These transverse slices were cut at right angles to the long axis of the heart^2^. This technique, for the first time, enables the use of a series of slices prepared from ventricle to measure transmembrane potential (Vm) and intracellular Ca^2+^ transient (CaT) with high temporal and spatial resolution in each slice. This allows profiling of the ventricular transmural and regional gradients in Vm and CaT and characterization of the transmural and regional profiles of action potential and CaT alternans associated with ventricular arrhythmias.

**Fig. 1:**
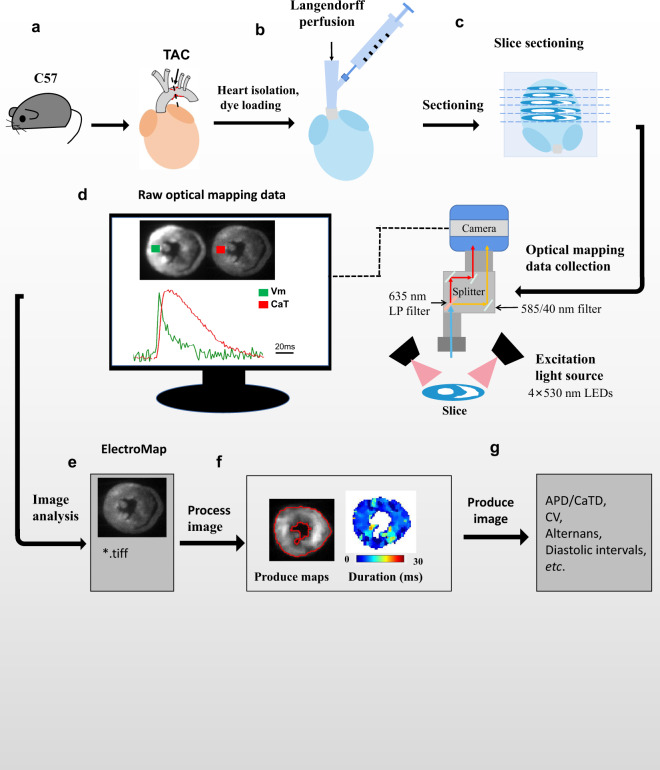
The schematic workflow of the assay design. (**a**) Constriction applied on the aortic arch between the innominate artery and left common carotid artery causes an increase in blood flow pressure through the stenosis. The extent to which the flow is decreased is proportional to the severity of the stenosis; (**b**) The isolated heart Langendorff perfusion, and fluorescent dyes loading been injected through the injection port near the cannula; (**c**) The heart was embedded in 4% low-melt agarose and cut into slices transversally at the thickness of 350 μm with vibratome; (**d**) Vm and CaT fluorescence signals were recorded by a custom-made optical mapping system. CaT fluorescence was collected using 585/40 nm bandpass filter while Vm emission was collected using a 662 nm longpass filter; (**e**) Raw data were loaded into ElectroMap; (**f**) Pre-process image; (**g**) Produce image. LP, longpass.

Such high-throughput transverse slice optical imaging technique thus provides a unique opportunity for exploring the mechanistic association of transmural electrical and intracellular Ca^2+^ remodelling in pathological hypertrophy and ventricular arrhythmogenesis that leads to SCD. To achieve this goal, a comprehensive dataset has been generated by experimentation and analysis using healthy murine hearts (defined as healthy control group *n* = 3) and pathological hypertrophic hearts *(n* = 3) induced by transversal aortic constriction (TAC). Figure 1 illustrates a schematic overview of the study design, from model generation, characterisation, living heart slicing, high-throughput optical imaging, data processing and analysis. The dataset has the following unique features. Firstly, a well-established TAC model allows the generation of reliable murine pathological hypertrophy mimicking pressure-overloaded hypertrophy in human condition. A high sensitivity echocardiography allows monitoring and quantifying the degree of the hypertrophy in living animals. Secondly, the use of mouse heart transverse slicing for high-throughput optical imaging allows study of electrophysiology of murine hypertrophic hearts in an organotypic pseudo two-dimensional model, permitting robust interrogation of Vm and CaT throughout the entire heart with exceptional regional precision and high spatio-temporal resolution. Thirdly, by using a sophisticated optical imaging analysis programme ElectroMap^2^, we are able to measure action potential duration (APD)/calcium transient duration (CaTD) at desired repolarization/reuptake percentage at each pixel across the tissue, diastolic interval, activation and conduction velocity (CV) were analysed, alternans and spontaneous firing and voltage-calcium latency. These comprehensive analysis allow (i) comparison of successive slices which form a stack representing the original geometry of the heart; (ii) profiling of transmural and regional gradients in Vm and CaT in the ventricle; (iii) characterization of transmural and regional profiles of action potential and CaT properties such as APD/CaTD, CV, alternans, diastolic intervals, etc. under stress (e.g., high frequency pacing or β-adrenergic stimulation) or hypertrophy condition.

Thus the dataset presented here offers a means for reuse to identify and understand hypertrophic transmural electrical and intracellular Ca^2+^ handling remodelling and associated ventricular arrhythmias in murine models. The approach also provides a basis for further use of drug testing and screening and for developing 2D and 3D computational models in the future.

## Methods

### TAC induced hypertrophic model

Cardiac hypertrophy was induced by means of thoracic aortic constriction (TAC) as described previously^3,4^ and according to the protocol approved by the Institution’s Animal Care and Use Committee of Southwest Medical University. In brief, 8-week-old C57 BL6/J male mice (Tengxin Biotechnology CO.,LTD, Chongqing, China) were subjected to TAC or without surgery after intraperitoneal injection of 1% pentobarbital. The mice underwent 3–4 mm sternotomy from the neck under spontaneous respiration. The transverse aortic arch was ligated between the innominate and left common carotid arteries with a 0.45 mm needle, and then the needle was removed, leaving a discrete region of stenosis. Echocardiography was conducted for assessing the cardiac hypertrophy at different time points following TAC.

### Echocardiography

Mice were anesthetized by inhalation of 1.5–2% isoflurane (RWD Life Science, Shenzhen, China). Transthoracic M-mode (short axis) echocardiographic recordings were performed using an echocardiography system equipped with RMV‐707B 44 MHz probe (Fujifilm Vevo 3100, VisualSonics, Toronto, ON, Canada) following a protocol described previously^5^. Measurements taken at end-systole and end-diastole were averaged to calculate intraventricular septal thickness (IVS), left ventricular anterior wall thickness (LVAW), left ventricular posterior wall thickness (LVPW), left ventricular internal diameter(LVID), ejection fraction (EF %) and fractional shortening (FS%). Maximal derived pressures were obtained during systole (dP/dtmax) and diastole (dP/dtmin) as indices representing cardiac contractile functions.

### Dye loading

Hearts were isolated under terminal anesthesia and cannulated via the aorta on the Langendorff system. Fluorescent dyes were loaded via the coronary circulation, applied by injection into the aortic cannula as described previously^2^. In brief, the Ca^2+^ dye Rhod-2 AM (Thermo Fisher Scientific, UK) was administered as a 50 µl bolus (stock solution: 1 mg/ml in dimethylsulfoxide) over a 5 min period, and recirculated for 20 min in the presence of 20% pluronic F127 (Invitrogen, Carlsbad, CA, United States) and the voltage sensitive dye RH237 (Thermo Fisher Scientific, UK) was delivered as a 30 μl bolus of 1 mM concentration. After dye loading, hearts were perfused with Krebs solution (7 g/L NaCl, 295 g/L KCl, 0.32 g/L MgSO_4_, 0.185 g/L NaH_2_PO_4_, 2.15 g/L NaHCO_3_, 1.98 g/L glucose, 1 mM CaCl_2_, bubbled with 95% O_2_ + 5% CO_2_ gas) containing 10 μM blebbistatin (Tocris Bioscience, Minneapolis, MN, United States), a myosin II inhibitor used to stop contractions and avoid movement artefacts.This step took 50–60 minutes.

### Slice preparation

Cardiac slices were prepared as described previously^2,6^. In brief, hearts were removed from the Langendorff set-up and were subsequently embedded in 4% low-melt agarose and cooled on ice at 4 °C. The specimen holder with agarose-embedded ventricles was mounted onto the stage of the vibratome filled with cold (4 °C) oxygenated (99.5% O_2_) Tyrode solution containing 2,3-butanedione 2-monoxime (BDM). Short-axis slices were cut at a thickness of 300 µm by vibratome (Leica VT1000s, Nussloch, Germany) with speed of 0.05 mm/s, amplitude of 1 mm and vibration frequency of 80 Hz. To prevent tissue from curling, slices were collected on Sylgard blocks and held in position using a nylon mesh in the preincubation chamber. The slice preparation, including slicing, harvest, and recovery took 60–70 minutes.

### Optical mapping

Slices were electrophysiologically assessed with the optical mapping method, using a custom-designed optical mapping system equipped with an electron multiplying charge-coupled devices (EMCCD) camera (Evolve 512, Photometrics, Tucson, AZ, United States). Figure 1 shows a schematic of the mapping set-up. Slices were kept in Krebs solution containing 10 µM blebbistatin at 37 °C during imaging. Four 530 nm light emitting diodes (LEDs) were used for excitation of the Ca^2+^-sensitive dye Rhod-2 and RH237. CaT fluorescence was collected using 585/40 nm bandpass filter while Vm emission was collected using a 662 nm longpass filter. Vm and CaT measurements were taken at maximal resolution (150 × 150 pixels; pixel area 100 × 100 µm) at a rate of 340 frames/sec. Slices were electrically stimulated with bipolar pulses of 2 ms duration, at voltages 1.5 times above threshold (5–10) and initial frequency of 2 Hz. For AP and CaT alternans investigations, slices were stimulated at 2, 4, 8 and 16 Hz frequency. The time duration of recording was less than one minute.

### Histological examination

After optical mapping, the slices were fixed for 24 h in 4% paraformaldehyde at room temperature, dehydrated by graded ethanol, and embedded in paraffin. Tissue sections (thickness of 5 μm) were deparaffinized with xylene, stained with hematoxylin and eosin (H&E), and viewed under a light microscope (Leica DM2000, Wetzlar, Germany).

### Data analysis

The raw datafiles (.tiff or .mat) were loaded into ElectroMap 1.0, a freely available MATLAB based software for analysis of electrophysiological datasets^7,8^. Manual region of interest selection was used to define the slice area and remove chambers from analysis. Images were pre-processed using a 3 × 3 pixel Gaussian filter with a standard deviation of 1.5. If baseline drift was observed, this was correcting using the top-hat filter correction method (filter length = 100 ms)^9^. To improve data quality, ensemble (multi-beat) averaging was applied to average the paced beats up to 8 Hz. Beats where temporally aligned by the maximum differential in the slice averaged voltage or calcium signal. This was not applied to the 16 Hz data due to significant alternans at this pacing cycle length^10,11^.

From the processed data, APD/CaTD were mapped across the slice surface as time from maximum depolarisation/upstroke to velocity to 75% (APD75/CaTD75) repolarisation/extrusion. Diastolic interval was defined as time from APD75 to the next AP activation (defined as the depolarization midpoint), which was also used to map activation^12,13^. From the activation maps, conduction velocity was calculated using a multi-vector methodology, where the local conduction speed and direction was calculated from a 5 × 5 pixel region using the polynomial method of Bayly *et al*.^14^. For alternans analysis, single beat AP/CaT amplitude was calculated and compared to the subsequent beat.

For dual analysis, the manually selected region on interest was applied to both voltage and calcium slice images. Voltage-calcium latency was measured as the time difference between the AP and CaT peak.

All data are shown as mean ± standard error, either from the whole heart (*n* = 3) or individual slices (11–17 slices per heart). Group mean were analyzed using two-tailed, paired or unpaired Student’s t-test or using One Way Analysis of Variance (ANOVA) with Tukey test as appropriate. Difference between categorical variables were analyzed using Fisher’s exact test. Statistical significance was accepted when *P* < 0.05.

## Data Records

The datasets have been made available from Figshare^15,16^.

The datasets consist of raw images (.tiff and .mat) of healthy control and TAC mice.

### Raw datasets: Optical mapping images of heart slices

‘Healthy control heart slices optical mapping RAW data’^15^ contains 14 slice images in .tiff format covering the C57BL/6 J wild type mouse heart. Images have been acquired, stitched and aligned as described in methods.

‘TAC heart slices optical mapping RAW data’^16^ contains 11 slice images in .tiff format covering the TAC mouse heart. Images have been acquired, stitched and aligned as described in methods.

### Processed datasets: analysed image stack and 3D data file

‘Processed Images’^15,16^ contains APD/CaTD, activation, diastolic interval and alternans images (two subsequent AP and CaT amplitude images at 16 Hz pacing).

A summary of these datasets is given in Table 1.

**Table 1:** A summary of files available from the online data repository^15,16^.

Subjects	Description	Format
Healthy control heart RAW data	3 hearts, 13–17 slices of optical mapping of TAC heart	.tiff and .mat
TAC heart RAW data	3 hearts, 13–17 slices of optical mapping of TAC heart	.tiff and .mat
Processed Images (APD75)	The APD75 of the raw images were analysed.	.tiff
Processed Images (CaTD75)	The CaTD75 of the raw images were analysed.	.tiff

## Technical Validation

**Fig. 2:**
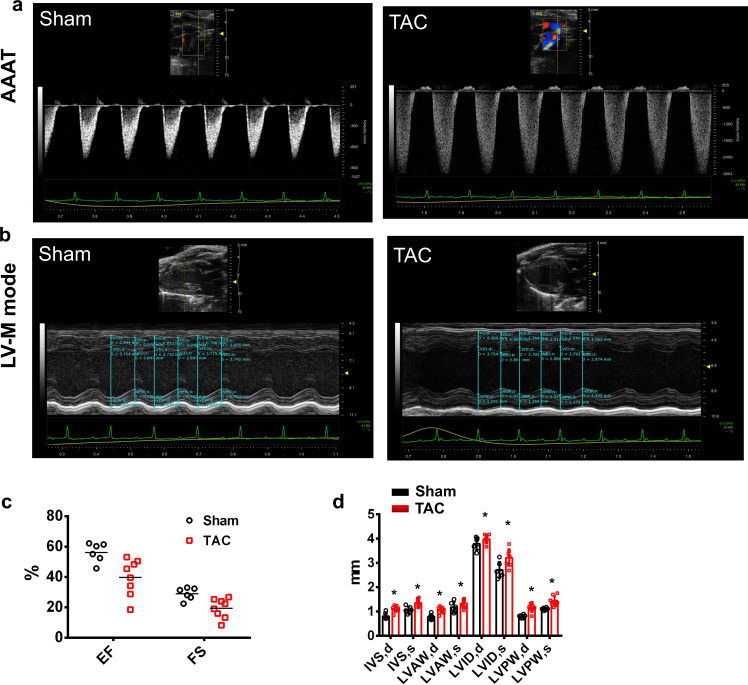
Echocardiographic and hemodynamic monitoring of cardiac function. Typical examples of cardiac function monitoring in mice without (healthy control, left column) or following TAC operation (right column). AAAT: echocardiographic evaluation of aortic arch acceleration time (AAAT). LV-M mode: M-mode echocardiographic recordings of left ventricular cycle. LV: intraventricular pressure measured in left ventricles (LV) with high-fidelity catheters. EF: ejection fraction (%); FS: fractional shortening (%); IVS,d: diastolic interventricular septal thickness(mm); IVS,s: systolic interventricular septal thickness(mm); LVAW,d: left ventricular anterior wall diastole thickness (mm); LVAW,s: left ventricular anterior wall systolic thickness (mm); LVID,d: left ventricular internal diameter in diastole (mm); LVID,s: left ventricular internal diameter in systole (mm); LVPW,d: left ventricular posterior wall thickness in diastole (mm); LVPW,s: left ventricular posterior wall thickness in systole (mm). *P < 0.05 and **P < 0.01, versus healthy control group. Unpaired Student’s t-test.

**Fig. 3:**
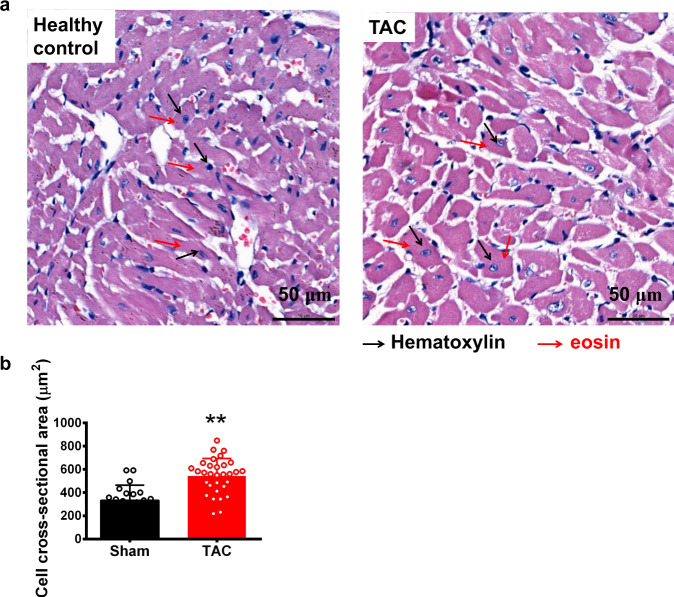
HE staining. (**a**) Hematoxylin and eosin (HE) staining of cross-sectional tissue slices of heart in the healthy control and TAC groups (×40). Scale bar: 50 μm. (**b**) Cross- sectional areas of myocyte in healthy control and TAC group. **P < 0.01, versus healthy control group, unpaired Student’s t-test.

To firstly validate TAC induced hypertrophy we applied echocardiographic imaging. Figure 2 demonstrates that, as expected, TAC reduced ejection fraction and fractional shortening. Histological analysis, Fig. 3, revealed increased cell cross sectional area.

**Fig. 4:**
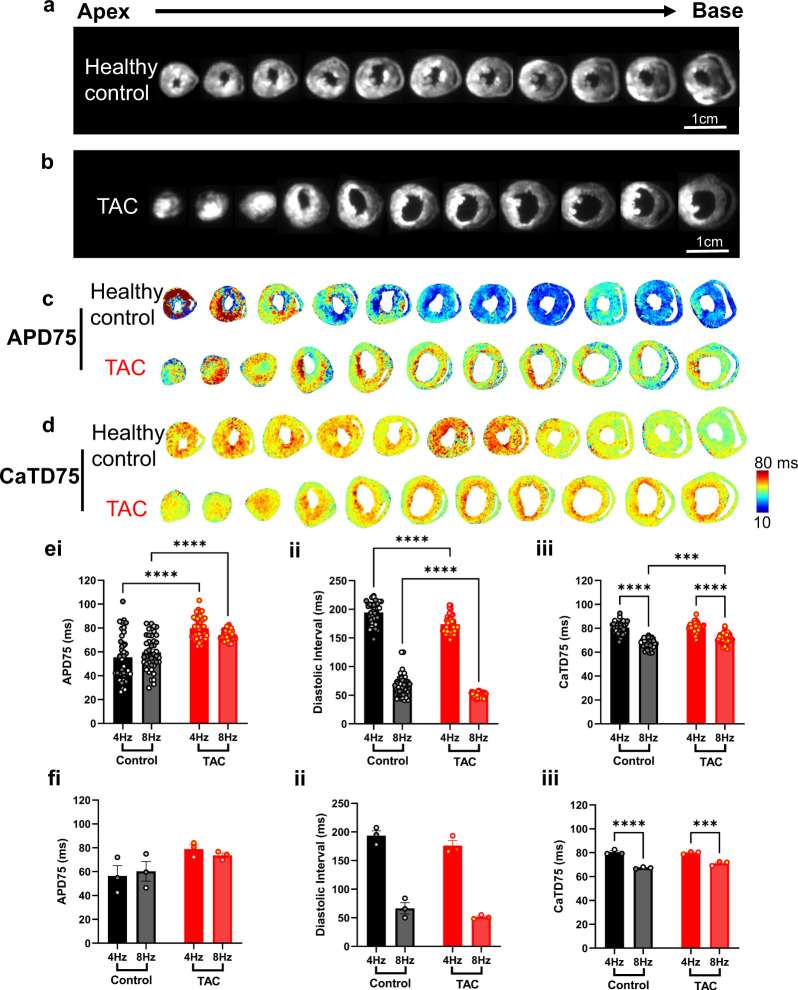
Analysis and characterization of the APD and CaTD of cardiac slices from TAC and healthy control hearts. (**a,b**) Fluorescence image (voltage-RH237) of transverse slices from apex to base of healthy control and TAC murine hearts, dual loaded with both transmembrane voltage (RH237) and intracellular calcium dyes (Rhod-2 AM); (**c,d**) The maps of APD75 and CaTD75 at 4 Hz pacing frequency (250 ms pacing cycle length), recorded from apex to base ventricular slices of healthy control and TAC murine hearts. (**e**) Grouped analysis of APD75, Diastolic Interval and CaTD75 in the healthy control and TAC slices. Each datapoint represents the mean from an individual slice. Slices from the same heart are shown with the same color. (**f**) Grouped analysis of APD75, diastolic interval and CaTD75 in the healthy control and TAC hearts. Each datapoint represents the mean from all slices collected for that heart. *n* = 3 hearts, 13–17 slices per heart. Statistical tests: e and f, One-way ANOVA with Tukey multiple comparisons. ***P < 0.001, ****P < 0.0001.

The data acquired by the transverse slice technique was then validated by studying the effects of i) pacing frequency and ii) TAC induced hypertrophy on key electrophysiological properties. Figure 4a,b shows the fluorescent images of dual dye loaded healthy control and TAC transverse slices. APD75 and CaTD75 were then mapped (Fig. 4c,d). At both 4 Hz and 8 Hz pacing frequency, TAC significantly prolonged APD75 in the transverse slices (Fig. 4e,i). For example, APD75 = 59.64 ± 1.44 ms in healthy control slices vs 74.23 ± 0.63 ms in TAC slices (8 Hz pacing, *P* < 0.0001). Diastolic interval showed an expected and concurrent reduction in response to TAC (Fig. 4e,ii). TAC also increased CaTD75 from 67.29 ± 0.60 ms in healthy control slices to 71.34 ± 0.60 ms in TAC slices (Fig. 4e,iii, 8 Hz pacing, *P* < 0.0001). CaTD75 also reduced in response to increased pacing frequency in both Sham and TAC slices, Fig. 4e,iii. These differences (other than pacing frequency reduction of CaTD75) were not observed when the data are expressed as means from the individual hearts included in this dataset, Fig. 4f.

**Fig. 5:**
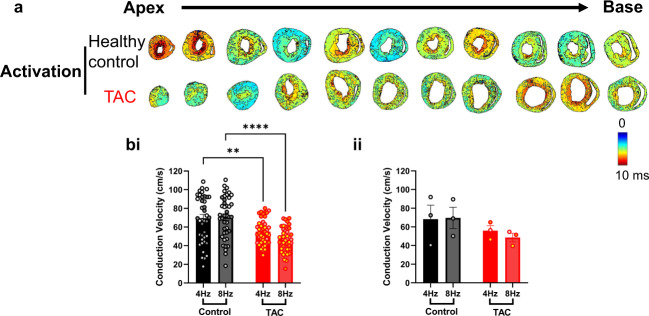
Activation maps of cardiac slices from healthy control and TAC hearts. (**a**) Ca^2+^ activation maps at 4 Hz pacing frequency (250 ms pacing cycle length) recorded from apex to base ventricular slices of healthy control and TAC heart. (**b**) Grouped analysis of conduction velocity in the healthy control and TAC slices (i) and hearts (ii). *n* = 3 hearts, 13–17 slices per heart. Statistical tests: b, One-way ANOVA with Tukey multiple comparisons. **P < 0.01, ****P < 0.0001.

Figure 5 shows the effects of pacing frequency and TAC induced hypotrophy on activation (Fig. 5a) and conduction velocity (Fig. 5b). At 8 Hz pacing, TAC significantly reduced conduction velocity from 64.89 ± 4.90 cm/s in healthy control slices to 46.61 ± 2.02 cm/s in TAC slices (Fig. 5b,i, *P* < 0.01). As with APD, CaTD and diastolic interval, conduction velocity differences were not observed when the data are expressed as means from the individual hearts, Fig. 5b,ii.

**Fig. 6:**
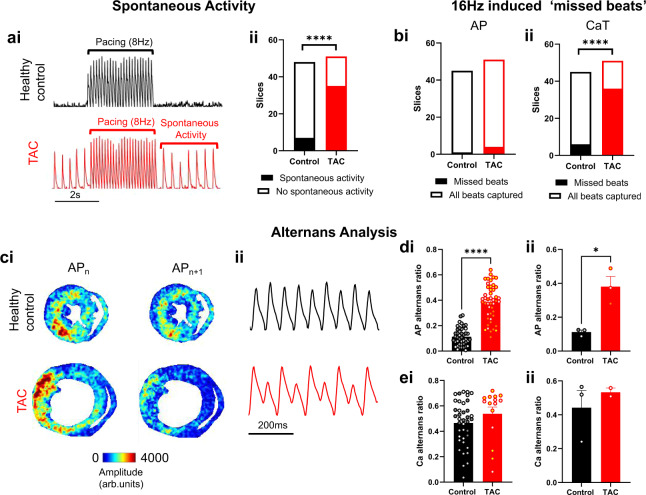
Arrythmia analysis. (**a**) Example voltage recoding from healthy control and TAC hearts before, during and after 8 Hz pacing (i) and grouped data showing the presence of spontaneous activity in the healthy control and Tac slices (ii). (**b**) Grouped analysis of missed action potentials (i) and calcium transients (ii) in healthy control and TAC slices. (**c**) Example amplitude maps for successive beats (i) and voltage recordings at 16 Hz pacing in healthy control and TAC slices. (**d**) Grouped analysis of AP alternans ratio in the healthy control and TAC slices (i) and hearts (ii). (**e**) Grouped analysis of CaT alternans ratio in the healthy control and TAC slices (i) and hearts (ii). *n* = 3 hearts, 13–17 slices per heart. Statistical tests: a and b, Fisher’s exact test. d and e, Student’s t-test. *P < 0.05, ****P < 0.0001.

Figure 6 summarises arrhythmic activity in the slices. Spontaneous activity was defined as consistent action potential firing at >2 Hz frequency when pacing was not applied, Fig. 6a,i. Spontaneous activity was present in a significantly greater number of TAC slices (35/51 slices) compared to healthy control slices (7/48 slices, *P* < 0.0001), Fig. 6a,ii. 16 Hz pacing induced pronounced arrhythmic activity including AP and CaT alternans and ‘missed beats’, where no AP/CaT was observed when pacing was applied. In many slices, this was observed as every other AP/CaT not firing, i.e. the effective cycle length remained at 8 Hz despite 16 Hz stimulation. A small number of slices (3 slices, all control healthy) also demonstrated fibrillation type signals, although the difficulty in discerning this behaviour from noise in the optical signals meant this was challenging to quantify. These slices where therefore removed from further arrythmia analysis. Fig. 6b,i shows a similar number of healthy control and TAC slices demonstrated missed APs. However, a significantly greater number of TAC slices displayed missed CaTs (36/51, vs 6/45 in healthy control slices, *P* < 0.0001), Fig. 6b,ii.

Figure 6c,i illustrates the mapping of alternans behaviour as AP amplitude of successive beats (AP_n_ and AP_n+1_). Example optical action potential recordings of this behaviour at specific pixel locations are shown in Fig. 6c,ii. Quantification of the AP alternans ratio revealed increased alternans behaviour in TAC slices compared to healthy control. Figure 6d. This was evident both when analysing individual slices (AP alternans ratio = 0.38 ± 0.02 vs 0.11 ± 0.01, TAC vs healthy control, *P* < 0.0001) and means (AP alternans ratio = 0.38 ± 0.06 vs 0.11 ± 0.01, TAC vs healthy control, *P* < 0.05) from all hearts. No differences where observed in CaT alternans ratio, Fig. 6e, although many TAC slices could not be used for this analysis due to the aforementioned ‘missed’ CaT behaviour.

**Fig. 7:**
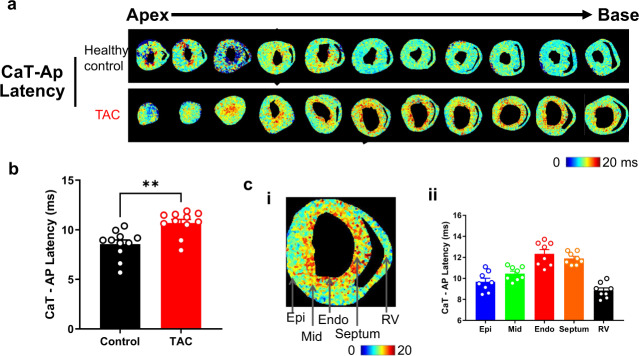
The CaT-AP latency of cardiac slices from healthy control and TAC hearts. (**a**) The maps of CaT-AP latency at 4 Hz pacing frequency (250 ms pacing cycle length) recorded from apex to base ventricular slices of healthy control and TAC heart; (**b**) Bar graph of CaT-AP latency of healthy control and TAC slices. (**c**) The CaT-AP latency of different regions of TAC slices. (i) The maps of CaT-AP latency at 2 Hz pacing frequency (500 ms pacing cycle length) from TAC ventricular slices; (ii) Bar graph of CaT-AP latency of different regions of TAC slices. Epi, Epicardium; Mid, Myocardium; Endo, Endocardium; RV, right ventricular. *n* = 11 slices compared with healthy control hearts. Statistical tests: B. Students t-test. **P < 0.01.

Dual loading of these slices enables analysis of voltage-calcium coupling, such as voltage-calcium latency. Figure 7a shows example analysis of CaT-AP latency from all slices in healthy control and TAC hearts. TAC slices displayed a prolonger CaT-AP latency, which is more pronounced at the endocardium compared to other ventricular areas. The pathophysiological significance of this preliminary analysis however requires further investigation.

### Limitations

The process of transverse slicing can possibly cause tissue injury by cutting perpendicular to fibre alignment in the heart. This may cause patrial uncoupling of cardiomyocytes, and so regional differences may be more pronounced. Our histological studies (Fig. 3) suggest that tissue injury in minimal, although this requires further investigation.

Ca^2+^-sensitive dyes, such as Rhod-2, in the cardiomyocytes can impact on calcium handling properties by chelating free Ca^2+^. This may in some part explain alternans and miss CaTs observed in this dataset. However, previous studies have shown that the dissociation constant of Rhod-2 (570–710 nM) has minimal impact on the Ca^2+^ transient, unlike other higher affinity Ca^2+^-sensitive dyes. Rhod-2 also displays favorable spectral properties for dual imaging with Rh237. However, it was noted that the RH237 signal can be weak when combining with Rhod-2. Some slices where therefore removed from the analysis and dataset. All slices included (Table 1) met minimum signal quality criteria that enhabled effective analysis.

## Data Availability

All data analysis was conducted using ElectroMap software with processing and analysis settings as described above. ElectroMap MATLAB code is available at https://github.com/CXO531/ElectroMap.
